# Support needs assessment tool for people with disability wanting to participate in sport and exercise (SNAT-SE): Usability and acceptability testing

**DOI:** 10.1016/j.jsampl.2025.100111

**Published:** 2025-07-14

**Authors:** Jessica Hill, Kelly Clanchy, Stewart Trost, Jennifer Fleming, Emma Beckman, Sean Tweedy, Iain Dutia, Sjaan Gomersall

**Affiliations:** a School of Health and Rehabilitation Sciences, The University of Queensland, Brisbane, Australia; b School of Health Sciences and Social Work, Griffith University, Gold Coast Australia; c The Hopkins Centre, Griffith University, Nathan, Australia; d School of Human Movement and Nutrition Sciences, The University of Queensland, Brisbane, Australia; e The Queensland Centre for Olympic and Paralympic Studies, The University of Queensland, Brisbane, Australia; f Australian Catholic University, School of Allied Health, Brisbane, Australia; g Health and Wellbeing Centre for Research Innovation, School of Human Movement and Nutrition Sciences, The University of Queensland, Brisbane, Australia

**Keywords:** Physical activity, Health promotion, Allied health, Coaching, Measurement

## Abstract

**Background:**

People with disability participate in sport and exercise at lower rates than the general population. Health and fitness professionals’ lack of knowledge regarding the support needs of people with disability has been identified as a major barrier to participation. To address this barrier, we developed a support needs assessment tool for people with disability wanting to participate in sport and exercise. The present study explored the usability and acceptability of the tool from the perspectives of the end-users.

**Method:**

An online survey was used to gather data on the usability and acceptability of the tool from the perspectives of people with disability, health professionals, community-based fitness professionals, and relevant researchers.

**Results:**

A total of 52 people completed the survey. Participants reported that the SNAT-SE was a useful and acceptable tool to assess the support needs of people with disability wanting to participate in sport and exercise. Participants also provided recommendations on refinements to further enhance the use of the tool. Refinements included increased clarity of the language used throughout the tool, a reduction in the overall length and flexibility in administration to reduce the time burden, and modifications to ensure all disability populations could equally benefit from the tool.

**Conclusion:**

Overall, the tool showed good usability and acceptability. Further research is required to evaluate the tool's effectiveness in improving the confidence and quality of service delivery of health and fitness professionals supporting people with disability to participate in sport and exercise.

**Key points:**

1. Previous literature has highlighted lack of health and fitness professional knowledge as a major barrier to people with disability participating in community sport and exercise.
2. To address this barrier, we developed a support needs assessment tool for people with disability wanting to participate in sport and exercise (SNAT-SE).
3. Overall, the SNAT-SE was deemed useful and acceptable by its end-users with further recommendations provided to improve its use in practice.
4. In response to participant recommendation's changes have been made and a final version of the SNAT-SE has been developed.

## 1 Introduction

According to the World Health Organisation, physical activity involves “any bodily movement produced by skeletal muscles thar require energy expenditure, including activities undertaken while working, playing, carrying out household chores, travelling and engaging in recreational pursuits” [1]. Exercise is a key form of leisure-time physical activity for individuals across the lifespan [2]. The primary distinction between exercise, and other forms of recreation or leisure-time physical activity is that exercise involves planned, structured and repetitive activities performed with an aim to improve fitness, in one or more body system [2]. Sport is considered a form of exercise and is defined as a physical activity “requiring high levels of exertion, physical athleticism, or dexterity which confer a benefit to physical health when practiced with sufficient frequency” ([2], page 5). For people with disability, a grey area in the distinction between recreation and leisure based physical activity and exercise exists, with this distinction depending on the health and training status of the individual [2]. For example, whilst for many going on an afternoon walk could be considered recreation, for some people with disability, the energy expenditure required to participate in this activity may constitute as exercise.

Like physical activity in general, people with disability have lower rates of sport and exercise participation when compared to the general population and are faced with many barriers to participation [[3], [4], [5], [6], [7], [8]]. Consistently, lack of fitness professional (i.e., coaches, trainers, instructors) knowledge has been identified as a major barrier with fitness professionals themselves recognising their lack the knowledge regarding how to effectively identify and address the support needs of people with disability [9,10]. For people with disability ‘supports’ needed to participate in meaningful activities can vary greatly regarding pattern and intensity [11]. Pattern of supports can include support from another person, equipment/assistive technology, changes to the environment, or modifications to the way an activity is performed [22]; whilst intensity refers to the frequency, duration and degree in which this support is required [23]. Support needs assessment tools are routinely used by health professionals, educators and employers to understand and address the support needs of people with a disability [12]. In addition to providing valuable information relating to the level of support the individual requires, support needs assessment tools can be used to facilitate communication and interprofessional collaboration between different professions [10,12]. Whilst several different support needs assessment tools exist addressing a wide range of activities (e.g., school participation, activities of daily living, employment), and populations (e.g., ages and disability groups), none specifically address participation in sport and exercise [10].

To address this gap, our research team developed the Support Needs Assessment Tool for people with disability wanting to participate in Sport and Exercise (SNAT-SE). The SNAT-SE was developed in four phases using a pragmatic participatory approach [13]. Phase four of this project is presented within this manuscript and aimed to evaluate the usability and acceptability of the SNAT-SE among its anticipated end-users. Within research, the concept of usability considers aspects of the perceived usefulness and ease of use of an innovation by the targeted end-users, where acceptability refers to the degree in which the innovation meets the needs of the target end-users, within their specific context [14]. Usability and acceptability testing assists researchers to identify how their innovation is received by its intended end-users, as well as provides them with the opportunity to gain important feedback, enabling improvements to be made ([14]). This iterative process assists to ensure that the final product is fit for purpose, enhancing its translation into practice ([14]).

## 2 Methods

### 2.1 Study design

A quantitative descriptive design was employed using an online survey (Ng et al., 2024). Ethics approval was obtained from (2024/HE000843).

#### 2.1.1 The support needs assessment tool for people with disability wanting to participate in sport and exercise (SNAT-SE)

As previously stated, the SNAT-SE was developed across four phases. In phase one, two qualitative studies were conducted with fitness professionals to explore their experiences working with people with disability [9,10]. Within this study, participants highlighted the lack of fitness professional knowledge regarding how best to support people with disability, and the need for educational tools and resources [9,10]. To address this need, in phase two, a scoping review was conducted to explore how the support needs of people with disability wanting to participate in physical activity, including sport and exercise were assessed [9]. Findings of this review revealed that although several general tools existed which included aspects of physical activity participation, none were specific to sport and exercise [9]. In phase three, an exploratory study was conducted to gain a holistic understanding of the support needs of people with disability participating in community sport and exercise [9]. Participants within this study described a dynamic relationship between managing their health condition/s with balancing the mental and physical energy available to complete their daily activities (e.g., dressing, eating, grooming, employment, etc), along with their sport and/or exercise participation.

Findings from the studies conducted in these first three phases resulted in the development of the pilot SNAT-SE (Supplementary Material 1). This tool consisted of 65-items addressing the domains of disability and health, participation in daily activities, and sport and exercise participation. The tool was designed to be completed by the health or community fitness professional during a consultation with their client with disability, or with a support person by proxy. Within the consultation, the health or fitness professional would be required to indicate on a 5-point Likert scale (1 indicating ‘no difficulty’ and 5 indicating ‘extreme difficulty/cannot do’) how much difficulty their client experienced with specific activities and skills due to their disability. The individual with disability was also provided with the option to identify if they required support from a person, equipment, or activity adaptation to perform each activity or skill. At the completion of the tool, the client would receive a score for each domain, as well as an overall score with higher scores indicating higher support needs. The aim of this score was to inform the health and fitness professionals how best to support their client, as well as who they might need to liaise with to provide the most effective client-centred care (e.g., higher scores within the domain of daily living activities might indicate a referral to an occupational therapist).

#### 2.1.2 Participants

Purposive sampling was used as potential participants were initially drawn from the professional networks of the research team. Snowball sampling was also used as individuals were encouraged to pass on information of the study to relevant contacts. This method was selected to ensure recruitment of participants who met the inclusion criteria of the study. Individuals were eligible to participate in the study if they were an adult (>18 years) and met at least one of the following criteria: 1) a health (e.g., physiotherapist, occupational therapist, exercise physiologist) and/or fitness professional (e.g., personal trainer, coach or fitness instructor), 2) a person with disability, or a support person of a person with a disability, 3) a researcher with experience with tool development in the fields of disability, physical activity and/or sport and exercise participation, and/or 4) a researcher with experience in physical activity and/or sport and exercise. To gain the perspectives of a wide range of potential future end-users of the tool, health and fitness professionals were eligible to participate irrespective of their previous experience working with people with disability. All participants were required to provide informed consent prior to participating and no participants were financially compensated for their time.

#### 2.1.3 Data collection

Participants were emailed a portable document format (PDF) of the pilot SNAT-SE to review before completing the survey. The questionnaire used within the survey was based on a review of the literature (e.g., [15]; Lo et al., 2018; [16]), as well as the experience of the whole research team. The initial survey was developed by the first author (JH- an occupational therapist and qualified personal trainer, with over 10 years clinical and seven years research experience supporting people with disability participate in physical activities including sport and exercise) and reviewed by the research team prior to testing.

A draft of the survey was tested and evaluated by three examiners independent to the project in relation to its usability and the relevance of questions to the research aims. Examiners included one exercise physiologist and one occupational therapist with experience supporting people with disability to participate in sport and exercise, and one person with disability with experience participating in sport and exercise. The draft survey was reviewed after each trial by ​JH to allow for necessary refinements. At the completion of this process the final survey comprised 35 items including both open and closed answer questions. Section one addressed nine demographic questions, whilst Section two asked 26 questions relating to the usability and acceptability of the SNAT-SE (Supplementary Material 2). All surveys were distributed through the online Qualtrics software [Qualtrics, Provo, UT] allowing for data to be collected anonymously.

### 2.2 Analysis

Quantitative data were downloaded from Qualtrics and analysed using Microsoft Excel [Version 16.63.1]. All participant responses were analysed together with each closed response question analysed for total frequency of responses. Open response questions were analysed using content analysis allowing for the identification of common words, ideas and themes [17,18]. As per the procedures outlined by Erlingsson and Brysiewicz [18], first author JH ​independently read 100 ​% of responses to familiarise herself with the data. Next, in line with the research aim, JH ​divided up participant responses into meaning units, which were provided with a code. From these codes JH ​generated themes and sub-themes. At the same time, researcher KC (an accredited exercise physiologist with 16 years’ experience supporting physical activity participation with clients with disability and chronic health conditions) independently reviewed participant responses to generate codes. Both researchers then met to discuss any discrepancies within these codes in relation to the themes and sub-themes generated by JH. Discussions occurred until consensus was met that the final themes and sub-themes were an accurate reflection of participant responses.

## 3 Results

A full description of participant demographics is provided in Table 1. A total of 52 participants completed the survey. Most (*n* ​= ​35) were female ranging from 18 to 68 years in age (M ​= ​35, SD ​= ​11.8). Participants included 30 allied health professionals, 12 fitness professionals, 10 people with disability, and three support people with disability. Additionally, two participants described themselves as researchers with expertise in tool development, and seven as researchers in physical activity and sport/exercise participation. Allied health and fitness professional participants had varying levels of experience within their profession ranging from 6 months to 40 years (M ​= ​7.5, SD ​= ​9). Similarly, participants also had varying levels of experience working with people with disability ranging from no experience to 20 years (M ​= ​5.8, SD ​= ​6.3). When asked how many clients with disability they were currently supporting to participate in sport and/or exercise responses ranged from none, to 50. (M ​= ​19, SD ​= ​13.3). Participants with disability identified participating in a range of different sporting and exercise activities with swimming (*n* ​= ​5) and gym (*n* ​= ​5) being identified most frequently.

**Table 1:** Participant demographics N = 52.

Item	n = 1
**Male**	17
**Female**	35
**Person with disability**	**10**
Cerebral palsy	6
Spinal cord injury	3
Thermoregulation difficulty	1
Guillain barre syndrome	1
Chronic inflammatory demyelinating polyneuropathy	1
Myopia	1
Hydrocephalus	1
Cerebral vision impairment.	1
Multiple sclerosis	1
**Sport participation**	
Yes	10
**Sports**	
Para rowing	**1**
Swimming	5
Frame running,	1
Seated throws (discus and shot put)	1
Gym (strength and conditioning)	4
Physiotherapy based exercise	1
Yoga	1
Sailing	1
Bushwalking	1
Handcycling	1
**Support person of a person with disability**	**3**
Intellectual disability	1
Cerebral palsy	1
Mobility difficulties	1
**Sport participation**	
Yes	2
No	1
**Sports**	
Cricket	1
Frame running	1
Seated throws (discus and shot put)	1
Gym (strength and conditioning)	1
**Allied health professional**	**30**
Exercise physiologist	23
Physiotherapist	3
Occupational therapist	4
**Researcher**	**9**
Tool development	2
Physical activity and sport participation	7
**Fitness professional**	**12**
Exercise scientist	5
Fitness instructor, e.g., personal trainer, yoga instructor, etc.	10
Elite athlete coach	2
Grassroots coach	3

Response frequencies for the closed response questions are reported in Table 2. Overall, most participants believed a tool such as the SNAT-SE would be useful to assist health and community fitness professionals assess the support needs of people with disability wanting to participate in sport and exercise. Regarding the SNAT-SE specifically, majority of participants perceived the tool to be comprehensive, clear, and easy to read and understand.

**Table 2:** Usefulness, usability and acceptability of the SNAT-SE.

Response	n = 1
**Usability**	
**Are the number of items covered: n** = **50**	
Too few	2
Too many	15
Far too many	1
About right	32
**The length of the tool is: n** = **49**	
Far too long	2
Too long	14
About right	33
**The clarity of each item is: n** = **49**	
Not clear at all	1
Mostly clear	18
Clear	21
Very clear	9
**How useful would a tool such as this be regarding assisting health and community-based fitness professionals to assess the support needs of people with disability wanting to participate in sport and exercise. n** = **46**	
Not helpful	2
Somewhat helpful	5
Helpful	22
Very helpful	17
**How easy is the tool to read? n** = **46**	
Difficult	1
Somewhat easy	14
Easy	17
Very easy	14
**How easy is the tool to understand? n** = **49**	
Difficult	1
Somewhat easy	3
Easy	29
Very easy	16
**Acceptability**	
**How comprehensive did you find the items included within the tool? n** = **49**	
Mostly comprehensive	10
Comprehensive	20
Very comprehensive	19
**The language used to describe each item is: n** = **49**	
Inappropriate	2
Neutral	4
Appropriate	28
Very appropriate	15
**How relevant did you find the items in relation to the sport and exercise participation of people with disability? n** = **49**	
Not relevant	1
Mostly relevant	7
Relevant	24
Very relevant	17
**How relevant did you find the items in relation to the sport and exercise participation of people with disability? n** = **49**	
Not relevant	1
Mostly relevant	7
Relevant	24
Very relevant	17
**Regarding the support needs for people with disability wanting to participate in sport and exercise, did the items seem: n** = **48**	
Biased towards specific health and/or fitness professionals	3
Biased towards specific support needs	7
Biased towards specific disability populations	5
Completely balanced	33
**Would you recommend this tool to others? n** = **47**	
Probably not	2
Maybe	10
Yes	25
Definitely	10

**Fig. 1:**
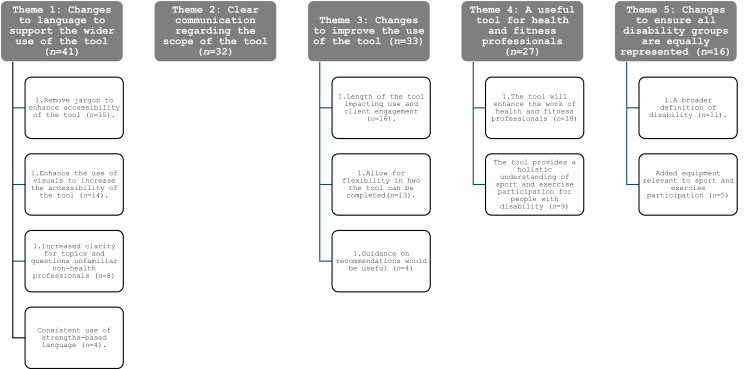
Themes and subthemes.

Content analysis of participant responses to the open response questions revealed five overarching themes, and 11 sub-themes (Fig. 1). Within these themes participants discussed both the benefits of the tool, as well as areas for refinement to enhance its use. Specific modifications made to the SNAT-SE are outlined in Table 3, and a full version of the revised tool can be found in Supplementary Material 3.

**Table 3:** Description of modifications made to the SNAT-SE in result of participant feedback.

Original tool	Relevant themes and subthemes	Summary of feedback	Modification made
Inclusion of jargon familiar to health and fitness professionals e.g., “mobilise”, “hydration”, etc.	Theme 1 – Subtheme 1.1	Removal of all jargon to ensure that the tool can be completed by people with disability and/or their support people.	Examples include, “mobilise” changed to “move around” and “hydration” changed to “drinking”.
Use of 11 ​pt font throughout the tool.	Theme 1 – Subtheme 1.2	Format and size of font may be difficicult for some people to see and interpret.	Two Likert scales using 48 ​pt font have been added to support accessibility to clients with disability and vision impairment.
White, grey and black colour contrast used throughout the tool.	Theme 1 subtheme 1.2	Lack of clear colour contrast might make it difficult for people with vision impairmant to complete.	The use of red, white and black colour contrast used throughout the tool.
No description of the relevance of daily activities to sport and exercise participation.	Theme 1 – Subtheme 1.3	Participants expressed confusion regarding the relevance of the daily living items to sport and exercise participation.	Information has been provided at the start of each domain to explain the relevence of the included items.
Deficit-focused languaged used throughout the tool e.g., “no difficulty”, “minor difficulty”, etc.	Theme 1 – Subtheme 1.4	Participants requested consistant use of strengths based language throughout the tool.	Examples of changes include “does not influence sport/exercise participation”, “slightly influences sport/exercise participaion" and “can easily do on my own”, “can do on my own with some difficulty”, etc.
No description of the purpose or scope of a needs assessment tool in relation to other forms of assessment.	Theme 2	Participants lacked clarity of the purpose and scope of a needs assessment tool.	The purpose and scope of the tool has been outlined within the ‘instructions for use’ in line with other forms of assessment.
65-Item tool addressing the domains of disability and health, participation in daily activities, and sport and exercise.	Theme 3 - subtheme 3.1	The length of the tool could impact the use of the tool. Questions included within the disability and health domain were often replicated within participation in daily activities, and sport and exercise making them redundant.	Removal of disability and health reducing the length of the tool to 57 items.
Designed to be completed by the health or fitness professional in consultation with the client with disability and their support person if required.	Theme 3 – Subtheme 3.2	The depth of information was useful to obtain, however the time it would take to complete the tool within a session could impact it's use.	Additional instuctions have been included to allow for the tool to be completed by the health and fitness professional in consultation with the client, or by the client with disability or their support person prior to the session.
No further guidance was provided relating to additional supports health or fitness professionals might access post completion of the tool.	Theme 3 – Subtheme 3.3	Participants were unsure where to seek futher assistance if their client was identified to have high support needs.	The addition of a referral table within the ‘instructions for use’ providing recommendations on who the health or fitness professional might collaborate with if their client scores highly within specific domains and/or items.
Few questions relating to people with psychosocial disability.	Theme 5 – Subtheme 5.1	Further items should be included to ensure all disabilities are represented.	Examples of additional items include, “managing your emotions”, “managing your mental health”, “motivating yourself to participate in sport/exercise”.
No sport specific equipment listed in equipment/aides.	Theme 5 – Subtheme 5.2	The ability to list sports specific equipment is required for some clients.	“Sports specific equipement (please list)” has been added to the domain of sport and exercise participation.

### 3.1 Theme 1: Changes to language to support the wider use of the tool

Several participants (*n* ​= ​41) discussed ways in which the language could be modified to support the broad use of the tool with a wide range of end-users. These changes fell under four subthemes, 1) removal of jargon to enhance the accessibility of the tool (*n* ​= ​15), 2) enhance the use of visuals to increase the accessibility of the tool (*n* ​= ​14), 3) increased clarity of topics that may be unfamiliar to non-health professionals (*n* ​= ​8), and 4) consistent use of strengths-based language (*n* ​= ​4). Within these subthemes, participants emphasised the importance of the tool being accessible to all health and fitness professionals, and all disability populations including those with a vision impairment, or an intellectual disability. Specific suggestions provided included the removal of medical ‘jargon’ (e.g., mobilise and sport/exercise history), as well as enhanced use of visual supports such as larger font size and greater colour contrast.

### 3.2 Theme 2: Clear communication regarding the scope of the tool

Several participant (*n* ​= ​32) responses indicated that further clarity regarding the purpose and scope of the tool was required. Several participants discussed the need for items that assessed individual performance components such as motor skills and communication. These responses highlighted the need for further clarity of the purpose of a support's needs assessment tool and how SNAT-SE was designed to be used in combination with other forms of assessment.

### 3.3 Theme 3: Changes to improve the use of the tool

Many participants (*n* ​= ​33) provided recommendations on how to improve the use of the tool when implemented within clinical and community settings. These changes fell under three subthemes, 1) length of the tool may impact use and client engagement (*n* ​= ​16), 2) allow for flexibility in how the tool can be completed (*n* ​= ​13), and 3) a need for more guidance for community-based fitness professionals (*n* ​= ​4). Within these subthemes, participants discussed the time restraints health and fitness professionals experience within their sessions and as a result the potential barriers they might face completing a tool such as the SNAT-SE. Several participants discussed the potential of the client completing the tool themselves prior to attending the first session, thus affording the health and/or fitness professional with the time to then have an in-depth discussion with their client on the specific support needs relevant to them.

### 3.4 Theme 4: A useful tool for health and fitness professionals

Many participants (*n* ​= ​27) perceived the SNAT-SE would be a useful tool to aid health and fitness professionals to support people with disability in sport and exercise. Some expressed that they wished the tool was available now. These benefits were discussed within two subthemes, 1) the tool will enhance the work of health and fitness professionals (*n* ​= ​18), and 2) the tool provides a holistic understanding of sport and exercise participation for people with disability (*n* ​= ​9). Within these two subthemes, participants expressed that they valued the ‘holistic understanding’ the tool provided regarding sport and exercise participation for people with disability, and perceived it had the potential to support fitness professionals to provide appropriately tailored services/programs, thus enhancing the client experience.

### 3.5 Theme 5: Changes to ensure all disability groups are equally represented

Some participants (*n* ​= ​16) made specific recommendations on how the tool could be improved to ensure it was useful to all people with disability with recommendations falling under two subthemes, 1) a broader definition of disability (*n* ​= ​11), and 2) added equipment relevant to sport and exercise participation (*n* ​= ​5). Within these subthemes, participants perceived that there was a large focus on physical and intellectual disability, and further items were required to ensure people all disability populations were equally represented.

## 4 Discussion

In this study, we found the SNAT-SE to exhibit a high level of usability and acceptability by the end-users. In the analysis of open-ended responses, participants endorsed that the tool would provide health and fitness professionals with a holistic understanding of their client and the factors that could influence their sport and exercise participation. This finding is important as fitness professionals have previously expressed their concern that they lacked the necessary resources to effectively work with people with disability, negatively impacting their confidence and willingness to work with them as clients [9,10]. Future research should evaluate the impact of this tool on fitness professional confidence and service delivery.

Although several positive features relating to the use of the tool were identified, additional recommendations were also provided with changes to the tool being made accordingly. Firstly, changes were made to the language used within the individual items to ensure it could be used by all end-users irrespective of past training. One example of this, was that increased clarity was provided regarding the relevance of daily activity questions. Past studies have identified that several barriers to sport and exercise participation for people with disability are experienced throughout their everyday life, before even getting to their sport or exercise activity (e.g., cognitive and physical fatigue involved in getting dressed in the morning) [9,19]. However, for many fitness professionals who have not received training, or had previous experiences working with people with disability, questions relating to activities of daily living may appear irrelevant. In response, information relating to the influence of daily activities and life responsibilities on sport and exercise participation of people with disability was added to the revised tool to provide this clarity. Further guidance was also provided within the ‘instructions for administration’ to outline the scope and purpose of the tool to assist health and fitness to understand the complexity of sport and exercise participation for people with disability. These changes are in line with past research that has found many health and fitness professionals lacked knowledge about the complexity of sport participation for people with disability [8,19,20]. This in turn often led to them focusing more on assessing and encouraging performance, rather than supporting sustained participation. Future research should look to explore the effectiveness of the revised SNAT-SE in broadening health and fitness professional understanding of the complexity of sport and exercise participation for people with disability and identify any further knowledge gaps needing to be addressed.

Changes were also made to reduce the length of the tool. Lengthy or time-consuming assessment tools are often identified within the literature to negatively influence overall usability [16,21]. As such, several items deemed redundant by participants were removed reducing its overall length. Further, all jargon was removed and strength-based language applied throughout. These changes will assist people with disability and/or their support person to complete the tool prior to meeting with their health or fitness professional with the aim of reducing the time burden within a paid consultation. Future research should compare the validity and agreement of the information gathered when the tool is self-completed by a person with disability versus when completed in consultation with a health or fitness professional.

In line with past literature [9,10], several fitness professionals expressed the need for further guidance and recommendations on how to support their client once completing the tool. Previously, community-based fitness professionals have advocated for improved processes to support interprofessional practice with relevant health professionals when the support needs of their client extend beyond their scope [9,10]. To address this need, guidance has been provided within the revised SNAT-SE relating to who fitness professionals may need to refer to and/or collaborate with if their client identifies as having high support needs within a particular domain or item (e.g., for a client who experiences high support needs to manage their social and emotional needs they might be referred to a psychologist). Additional research is required to evaluate the effectiveness of this strategy. Finally, changes were made to ensure that the tool adequately captured the support needs of all people with disability, including those with psychosocial and/or sensory disability.

### 4.1 Strengths and limitations

This study had several strengths. Firstly, end-users were represented from a range of professions, disability groups, and sports. Gaining feedback directly from the end-users allowed for critical improvements to the SNAT-SE to be made ensuring that it is fit for purpose and can be translated into practice. The use of open-response questions in combination with closed response questions further enabled for deeper exploration of participants perception of the tool, aiding these improvements. Despite these strengths, several limitations remained. Firstly, snowball sampling was used. This may result in potential biases, and reduced generalisability of the findings as those with specific interests in the topic are more likely to be provided with information relating to the study and opt into participating. The sample size was small, and although attempts were made to recruit participants from a range of professional backgrounds and disability groups, there was a high representation of allied health professionals, in particular exercise physiologists. Of the participants with disability, there was a high representation of people with physical and neurological disability with people with psychosocial disability not being represented. Further, participants from culturally and linguistically diverse backgrounds were not specifically recruited meaning the usability and acceptability of the tool with this population was not evaluated. To address these limitations, thus improving the translation of the tool further research is required to evaluate its use with a more diverse sample to ensure the changes made are useful and acceptable by all potential end-users.

## 5 Conclusion

The SNAT-SE was described/rated as acceptable and could be used within practice to assist health and fitness professionals support people with disability participate in community sport and exercise. Based on end-user recommendations, refinements were made to improve the clarity of the wording used to describe the purpose and scope of the tool, and within individual items. Modifications were also made to reduce the length of the SNAT-SE and provide more flexibility on how the tool could be completed. Finally, additions were made to allow for a broader understanding of disability, ensuring that the support needs of all disability populations were addressed. Further research is required to determine if the revised SNAT-SE is effective in improving the confidence and quality of service delivery of health and fitness professionals supporting people with disability participate in sport and exercise.

## Declaration of authorship

The authors acknowledge that each author has read and approved the contents of this article.

## Funding

SRG is partly funded by the Health and Wellbeing Centre for Research Innovation (HWCRI), which is co-funded by The University of Queensland and Health and Wellbeing Queensland. No additional funding was received in the completion of this project.

## Declaration of competing interest

None.
