# Supplementary material for: Support needs assessment tool for people with disability wanting to participate in sport and exercise (SNAT-SE): Usability and acceptability testing
Source: JSAMS Plus. 2025 Jul 14;6:100111. doi: 10.1016/j.jsampl.2025.100111 (PMC13008460; doi:10.1016/j.jsampl.2025.100111)
Supplement: Multimedia component 1 [file mmc1.docx]

***Supplementary Material 1. Draft needs assessment tool***

**The support needs assessment tool for people with disability wanting to participate in sport and exercise (SNAT-SE) – Instructions for use**

The SNAT-SE is a 65-item tool addressing the domains of **Disability and Health**, **Participation in Daily Activities**, and **Sport and Exercise Participation**. The SNAT-SE is designed to be completed by a health or community-based fitness professional during a 60-minute consultation with their client. As the tool is designed to support the needs of all people with disability, the SNAT-SE can be completed with the client themselves, or with their support person acting as a proxy. Within all three domains, the fitness professional will be required to ask their client to indicate on a 5-point Likert scale (1 indicating ‘no difficulty’ and 5 indicating ‘extreme/cannot do’) how much difficulty they experience with specific activities and skills due to their disability. Further, clients will also be provided with the option to identify if they require support from a person, equipment or activity adaptation when answering each item. After completing the assessment tool, the client will receive a score for each domain, as well as an overall score with higher scores indicating higher support needs. These scores will assist to inform the health and community-based fitness professional how best to support their client, as well as who they may need to liaise with to provide the most effective client-centred care (e.g., higher scores within the domain of daily activities might indicate a referral to an occupational therapist).

*Demographic and background information*

| **C1** | **Name** |
| --- | --- |
|  | *Please provide response here* |
| **C2** | **Gender** |
|  | *Please provide response here* |
| **C3** | **Date of birth** |
|  | *Please provide response here* |
| **C4** | **If this assessment is being completed by a support person, please provide the name of person completing assessment** |
|  | *Please provide response here* |
| **C5** | **If this assessment is being completed by a support person, please identify the relationship to person (e.g., parent, guardian, support worker, etc).** |
|  | *Please provide response here* |
| **C6** | **Sport/exercise history in years.** |
|  | *Please provide response here* |
| **C7** | **What sport/exercise activities have you previously participated in, and at what level (e.g., participation/recreation, club, performance/nationals, etc.?** |
|  | *Please provide response here* |
| **C8** | **For the sport/exercise activities you identified within question C6, when did you last participate in these?** |
|  | *Please provide response here* |
| **C9** | **For the sport/exercise activities you identified within question C6, what was your experience of these, e.g., positive, or negative.** |
|  | *Please provide response here* |
| **C10** | **What sport/exercise activities would you like to participate in?** |
|  | *Please provide response here* |
| **C11** | **Why do you want to participate in sport/exercise?** |
|  | *Please provide response here* |
| **C12** | **How frequently would you like to participate in sport/exercise?** |
|  | *Please provide response here* |
| **C13** | **For how long you would like to participate in sport/exercise per session?** |
|  | *Please provide response here* |
| **C14** | **What is your preferred intensity of sport and exercise (light, moderate or vigorous)?** |
|  | *Please provide response here* |
| **C15** | **What time of day would it be best for you to participate in sport/exercise? AND why?** |
|  | *Please provide response here* |

*Disability and health*

| **H1** | **Please list your disability/health/medical condition/s.** | | | | | | | |
| --- | --- | --- | --- | --- | --- | --- | --- | --- |
|  | *Please provide response here* | | | | | | | |
| **H2** | **Do you experience, or are at risk of experiencing any of the following?** | | | | | | | |
|  | - Seizures - Pain - Fatigue - Hypertonicity (high tone) - Hypotonicity (low tone) - Pressure sores - Subluxation/dislocation - Aspiration - Depression - Anxiety - Hearing impairment - Vision impairment - Other (please state) | | | | | | | |
|  | **The following questions will ask about difficulties you might experience due to your disability/health/medical condition. You will also be asked to identify any supports you require in relation to your disability/health/medical condition. How much difficulty do you experience.** | **1**  **No difficulty** | **2**  **Minor difficulty** | **3**  **Moderate difficulty** | **4**  **Great difficulty** | **5**  **Extreme/**  **Cannot do** | **Support from a person** | **Support from Equipment** |
| **H3** | **Seeing things at a distance.** |  |  |  |  |  |  |  |
| **H4** | **Seeing things close.** |  |  |  |  |  |  |  |
| **H5** | **Hearing what people say to you or sounds around you.** |  |  |  |  |  |  |  |
| **H6** | **Communicating with familiar people.** |  |  |  |  |  |  |  |
| **H7** | **Communicating with unfamiliar people.** |  |  |  |  |  |  |  |
| **H8** | **Getting along with others.** |  |  |  |  |  |  |  |
| **H9** | **Managing your emotions.** |  |  |  |  |  |  |  |
| **H10** | **Mobilising on flat ground.** |  |  |  |  |  |  |  |
| **H11** | **Mobilising on uneven ground** |  |  |  |  |  |  |  |
| **H12** | **Using your hands to complete activities.** |  |  |  |  |  |  |  |
| **H13** | **Mobilising up and down stairs.** |  |  |  |  |  |  |  |
| **H14** | **Remembering and/or concentrating.** |  |  |  |  |  |  |  |
| **H15** | **Problem solving.** |  |  |  |  |  |  |  |
| **H16** | **Understanding what people say to you.** |  |  |  |  |  |  |  |
| **H17** | **Managing your sensory environment.** |  |  |  |  |  |  |  |
| **H18** | **Additional information regarding your disability/health/medical condition/s. *Please also use this section to provide further information on supports required.*** | | | | | |  |  |
|  | *Please provide response here* | | | | | |  |  |

*Daily activities*

| **D1** | **Over the last month, how has your disability/health/medical condition limited your ability to participate in your daily activities?** | **1**  **Not at all** | **2**  **Rarely** | **3**  **Weekly** | **4**  **Daily** | **5**  **Unable to participate** |  | | |
| --- | --- | --- | --- | --- | --- | --- | --- | --- | --- |
|  |  |  |  |  |  |  |  |  |  |
|  | **The following questions will ask about any difficulties you may have experienced participating in your daily activities. For each item you will also be asked to identify any supports you require to facilitate your participation. In the past month, how much difficulty did you have with:** | **1**  **No difficulty** | **2**  **Minor difficulty** | **3**  **Moderate difficulty** | **4**  **Great difficulty** | **5**  **Extreme/**  **Cannot do** | **Support from a person** | **Support from Equipment** | **Activity adaptation** |
| **D2** | **Getting where you need to go.** |  |  |  |  |  |  |  |  |
| **D3** | **Using the toilet.** |  |  |  |  |  |  |  |  |
| **D4** | **Cleaning yourself.** |  |  |  |  |  |  |  |  |
| **D5** | **Grooming activities.** |  |  |  |  |  |  |  |  |
| **D6** | **Dressing and undressing yourself.** |  |  |  |  |  |  |  |  |
| **D7** | **Managing nutrition, e.g., preparing and eating regular meals.** |  |  |  |  |  |  |  |  |
| **D8** | **Managing hydration.** |  |  |  |  |  |  |  |  |
| **D9** | **Managing your health e.g., taking medication.** |  |  |  |  |  |  |  |  |
| **D10** | **Organising self e.g., organising belongings before leaving the house.** |  |  |  |  |  |  |  |  |
| **D11** | **Completing household tasks e.g., cleaning, laundry.** |  |  |  |  |  |  |  |  |
| **D12** | **Using transport.** |  |  |  |  |  |  |  |  |
| **D13** | **Managing your employment and/or education.** |  |  |  |  |  |  |  |  |
| **D14** | **Accessing the community.** |  |  |  |  |  |  |  |  |
| **D15** | **Sleeping.** |  |  |  |  |  |  |  |  |
| **D16** | **Managing life responsibilities e.g., caring for a child or pet, attending appointments.** |  |  |  |  |  |  |  |  |
| **D17** | **Using technology e.g., telephone, computer, etc.** |  |  |  |  |  |  |  |  |
| **D18** | **Do you use any of the following equipment/aides to participate in your daily activities?** | | | | | | | | |
|  | - None - Cane/stick - Crutches - Orthoses - Splint - Manual wheelchair - Electric wheelchair - Pressure relief cushion. - Protheses – lower limb - Protheses – upper limb - Rollators - Standing frame - Walking frame - Therapeutic footwear - Glasses - Hearing aide - White cane - Assistance animal. - Augmentative and alternative communication - Others (please list) | | | | | | | | |
| **D19** | **Additional information regarding the supports you require to complete your daily activities. *Please also use this section to provide further information on supports required.*** | | | | | | | | |
|  | *Please provide response here* | | | | | | | | |

*Sport/exercise participation*

| **S1** | **Over the last month how has your disability/health/medical condition limited your ability to participate in sport/exercise?** | **1**  **Not at all** | **2**  **Rarely** | **3**  **Weekly** | **4**  **Daily** | **5**  **Unable to participate** |  | | |
| --- | --- | --- | --- | --- | --- | --- | --- | --- | --- |
|  |  |  |  |  |  |  |  |  |  |
|  | **The following questions will ask about any difficulties you may have experienced participating in sport/exercise. For each item you will also be asked to identify any supports you require to facilitate your participation. In the past month, how much difficulty did you have with:** | **1**  **No difficulty** | **2**  **Minor difficulty** | **3**  **Moderate difficulty** | **4**  **Great difficulty** | **5**  **Extreme/**  **Cannot do** | **Support from a person** | **Support from Equipment** | **Activity adaptation** |
| **S2** | **Accessing facilities e.g., gyms, sport fields.** |  |  |  |  |  |  |  |  |
| **S3** | **Managing the environment of facilities e.g., physical, sensory, etc.** |  |  |  |  |  |  |  |  |
| **S4** | **Mobilising around facilities.** |  |  |  |  |  |  |  |  |
| **S5** | **Participating in programs e.g., grass-roots sports, fitness classes.** |  |  |  |  |  |  |  |  |
| **S6** | **Using sport/exercise equipment e.g., racquets, balls, exercise machines etc.** |  |  |  |  |  |  |  |  |
| **S7** | **Engaging with others and joining group activities.** |  |  |  |  |  |  |  |  |
| **S8** | **Communicating with staff/coaches/teammates.** |  |  |  |  |  |  |  |  |
| **S9** | **Advocating for my own needs.** |  |  |  |  |  |  |  |  |
| **S10** | **Performing specific sport/exercise movement/skills.** |  |  |  |  |  |  |  |  |
| **S11** | **Accessing and managing funding required to participate in sport/exercise.** |  |  |  |  |  |  |  |  |
| **S12** | **Do you use any of the following equipment/aides to participate in sport/exercise?** | | | | | | | | |
|  | - None - Cane/stick - Crutches - Orthoses - Splint - Manual wheelchair - Electric wheelchair - Pressure relief cushion. - Protheses – lower limb - Protheses – upper limb - Rollators - Standing frame - Walking frame - Therapeutic footwear - Glasses - Hearing aide - White cane - Assistance animal - Augmentative and alternative communication - Others (please list) | | | | | | | | |
| **S13** | **Additional information regarding the supports you require to participate in sport/exercise. *Please also use this section to provide further information on supports required.*** | | | | | | | | |
|  | *Please provide response here* | | | | | | | | |
