# Supplementary material for: Support needs assessment tool for people with disability wanting to participate in sport and exercise (SNAT-SE): Usability and acceptability testing
Source: JSAMS Plus. 2025 Jul 14;6:100111. doi: 10.1016/j.jsampl.2025.100111 (PMC13008460; doi:10.1016/j.jsampl.2025.100111)
Supplement: Multimedia component 2 [file mmc2.docx]

***Supplementary Material 2: Survey tool***

*Part 1: Demographic information*

| 1. **Please state your gender** |
| --- |
|  |
| 1. **Please write your age in years** |
|  |
| 1. **Regarding this project, please select from the following which most appropriately describes your expertise. (Please select all that apply)** |
| - Physiotherapist (Skip logic to question 4). - Exercise physiologist (Skip logic to question 4). - Exercise scientist (Skip logic to question 4). - Occupational therapist (Skip logic to question 4). - Grass-roots coach (Skip logic to question 4). - Elite athlete coach (Skip logic to question 4). - Fitness instructor/trainer e.g., personal trainer, yoga instructor (Skip logic to question 4). - Person with disability (Skip logic to question 7) - Support person of a person with disability (Skip logic to question 7). - Researcher/academic with tool development expertise relevant to disability, physical activity, and/or sport and exercise (Skip logic to question 9). - Researcher/academic in the fields of physical activity, sport and/or exercise participation (Skip logic to question 9). - Other (please state). |
| 1. **How many years have you worked in your profession?** |
|  |
| 1. **How many years have you supported people with disability to participate in sport or exercise?** |
|  |
| 1. **Approximately how many clients with disability would you work with to support their sport and/or exercise participation?** |
|  |
| 1. **Please list your, or the person you support primary disability, as well as any co-occurring health/medical conditions.** |
|  |
| 1. **Do you, or the person you support currently participate in sport or exercise?** |
| - Yes (please list) - No |
| 1. **Please provide a brief description of your experience/expertise with tool development relevant to physical activity, and/or sport and exercise participation.** |
|  |

*Part 2: Tool specific questions.*

| **Please answer all following questions in relation to the draft support needs assessment tool for people with disability wanting to participate in sport and exercise.** |
| --- |
| 1. **Are the number of items covered:** |
| 1. **Far too few.** 2. **Too few.** 3. **About right.** 4. **Too many.** 5. **Far too many.**   **Additional comment:** |
| 1. **How relevant did you find the items in relation to the sport and exercise participation of people with disability?** |
| 1. **Not relevant at all.** 2. **Not relevant.** 3. **Mostly relevant.** 4. **Relevant.** 5. **Very relevant.**   **Additional comments:** |
| 1. **How comprehensive did you find the items included within the tool?** |
| 1. **Not comprehensive at all.** 2. **Not comprehensive.** 3. **Mostly comprehensive.** 4. **Comprehensive** 5. **Very comprehensive.**   **Additional comments.** |
| 1. **The length of the tool is:** |
| 1. **Far too short.** 2. **Too short.** 3. **About right.** 4. **Too long.** 5. **Far too long.**   **Additional comments:** |
| 1. **The clarity of each item is:** |
| 1. **Not clear at all.** 2. **Unclear.** 3. **About right.** 4. **Mostly clear.** 5. **Very clear.**   **Additional comment:** |
| 1. **The language used to describe each item is:** |
| 1. **Not appropriate at all.** 2. **Inappropriate.** 3. **Neutral.** 4. **Appropriate.** 5. **Very appropriate.**   **Additional comment:** |
| 1. **Regarding the support needs for people with disability wanting to participate in sport and exercise did the items seem:** |
| 1. **Biased towards specific disability populations.** 2. **Biased towards specific activities.** 3. **Biased towards specific support needs.** 4. **Biased towards specific health and/or fitness professionals.** 5. **Completely balanced.**   **Additional comment:** |
| 1. **Was any of the information new to you?** |
| 1. **All** 2. **Most** 3. **Some** 4. **Little** 5. **None**   **Additional comment:** |
| 1. **How useful would a tool such as this be regarding assisting health and community-based fitness professionals to assess the support needs of people with disability wanting to participate in sport and exercise.** |
| 1. **Not helpful at all.** 2. **Not helpful** 3. **Somewhat helpful.** 4. **Helpful.** 5. **Very helpful.**   **Additional comment:** |
| 1. **Would you recommend this tool to others** |
| 1. **Definitely not.** 2. **Probably not.** 3. **Maybe.** 4. **Yes.** 5. **Definitely**   **Additional comment:** |
| 1. **How simple is the tool to navigate?** |
| 1. **Very difficult.** 2. **Difficult.** 3. **Somewhat easy.** 4. **Easy.** 5. **Very easy.**   **Additional comment:** |
| 1. **How easy is the tool to read?** |
| 1. **Very difficult.** 2. **Difficult.** 3. **Somewhat easy.** 4. **Easy.** 5. **Very easy.**   **Additional comment:** |
| 1. **How easy is the tool to understand?** |
| 1. **Very difficult.** 2. **Difficult.** 3. **Somewhat easy.** 4. **Easy.** 5. **Very easy.**   **Additional comment:** |
| 1. **Please provide any additional thoughts/comments/or feedback you might have in relation to our draft tool.** |
|  |
