# Supplementary material for: Support needs assessment tool for people with disability wanting to participate in sport and exercise (SNAT-SE): Usability and acceptability testing
Source: JSAMS Plus. 2025 Jul 14;6:100111. doi: 10.1016/j.jsampl.2025.100111 (PMC13008460; doi:10.1016/j.jsampl.2025.100111)
Supplement: Multimedia component 3 [file mmc3.docx]

***Supplementary Material 3: The support needs assessment tool for people with disability wanting to participate in sport and exercise (SNAT-SE) - Revised***

**Instructions for administration**

The SNAT-SE is a 57-item tool addressing factors of the person (i.e., disability and health, motivations), and their participation in **Sport and Exercise,** and related activities. The SNAT-SE is designed to be used with adult clients (over the age of 18 years) and completed by a health (e.g., physiotherapist, exercise physiologist, etc) or fitness professional (e.g., personal trainer, coach, fitness instructor) during a client consultation, or by the client prior to the consultation (with or without assistance of a support person). Only sections relevant to the client are required to be completed. Therefore, depending on the level of detail obtained the SNAT-SE is expected to take between 60 to 90 minutes to complete. This can be achieved across multiple sessions if required.

The SNAT-SE has two domains: participation in daily activities, and sport and exercise. Within the domain of participation in **daily activities**, the client will be asked to indicate on a five-point Likert scale the level of impact each activity has on their ability to participate in sport and exercise. Within the domain of **sport and exercise** the client will then be asked to indicate on a 5-point Likert scale how much support they require to participate in specific activities, tasks and skills due to their disability. Within both domains, clients who indicate that they require support will be provided with the opportunity to identify what specific support they require (i.e., support from a person, equipment or activity adaptation). ***Please note that the SNAT-SE is a needs assessment tool only and is not intended to replace other forms of information gathering and assessment (e.g., client interviews, specific physical assessments, sport specific assessments). It is instead intended to provide guidance to the health and fitness professional on the pattern and intensity of supports their client might require to facilitate successful and sustained participation in sport and/or exercise.***

After completing the SNAT-SE, the client will receive a score for each domain, as well as an overall score, with higher scores indicating higher support needs. These scores will assist to inform the health and/or fitness professional how best to support their client, as well as who they may need to liaise with to provide those supports. For clients who receive a moderate to high score overall, or within the individual domains it is recommended that you refer and/or liaise with the appropriate health professional. Specifically, for clients who indicate requiring moderate to significant support (i.e., 4 or a 5) for any of the below items it is recommended that you refer to and/or liaise with the following health professionals.

| **Physiotherapy** | S17-S24 |
| --- | --- |
| **Exercise physiology** | S17-S24 |
| **Occupational therapy** | D1–D10, S3-S25 |
| **Speech and language pathology** | D5, S2, S9—S12 |
| **Audiologist** | S2 |
| **Psychology** | D8, S3, S4, S5-S8 |
| **Optometrist** | S1 |

**Support Needs Assessment Tool for people with disability wanting to participate in Sport and Exercise (SNAT-SE)**

***Demographic and background information***

| **C1** | **Name** |
| --- | --- |
|  | *Please provide response here* |
| **C2** | **Gender** |
|  | *Please provide response here* |
| **C3** | **Date of birth** |
|  | *Please provide response here* |
| **C4** | **If this assessment is being completed by a support person, please provide the name of person completing assessment.** |
|  | *Please provide response here* |
| **C5** | **If this assessment is being completed by a support person, please identify the relationship to person (e.g., parent, guardian, support worker, etc).** |
|  | *Please provide response here* |
| **C6** | **Please list your disability/health/medical condition/s.** |
|  | *Please provide response here* |
| **C7** | **Do you experience, or are at risk of experiencing any of the following?** |
|  | - Seizures - Pain - Fatigue - Hypertonicity (high tone) - Hypotonicity (low tone) - Pressure sores - Subluxation/dislocation - Aspiration - Depression - Anxiety - Hearing impairment - Vision impairment - Cognitive or behavioural difficulties - Fainting - Other (please state) |
| **C8** | **How long have you been participating in sport and/or exercise?** (please answer in number of years – if this is your first time participating in sport/exercise please write 0). |
|  | *Please provide response here* |
| **C9** | **What sport/exercise activities do you currently/have you previously participated in, and at what level** e.g., participation/recreation, club, performance/nationals, etc? |
|  | *Please provide response here* |
| **C10** | **For the sport/exercise activities you identified within question C9, what was your experience of these,** e.g., positive, or negative? |
|  | *Please provide response here* |
| **C11** | **What is the current frequency and duration of your sport and exercise participation** i.e., days per week and length of session? |
|  | *Please provide response here* |
| **C12** | **What sport/exercise activities would you like to participate in?** |
|  | *Please provide response here* |
| **C13** | **Why do you want to participate in sport/exercise?** |
|  | *Please provide response here* |
| **C14** | **How frequently would you like to participate in sport/exercise?** |
|  | *Please provide response here* |
| **C15** | **For how long you would like to participate in sport/exercise per session?** |
|  | *Please provide response here* |
| **C16** | **What is your preferred intensity of sport and exercise** i.e., light (e.g., fishing, stretching), moderate (e.g., brisk walking, dance, water aerobics) or vigorous (e.g., running, swimming, weight training)**?** |
|  | *Please provide response here* |
| **C17** | **What time of day would it be best for you to participate in sport/exercise? AND why?** |
|  | *Please provide response here* |
| **C18** | **How would you like to communicate with your health/fitness professional** e.g., Auslan, visual supports, demonstration, verbal only, etc? |
|  | *Please provide your response here* |

***Participation in daily activities***

For many people with disability, participation in their daily activities can influence their sport and exercise participation. For example, the cognitive fatigue a person experiences managing their life responsibilities, or the physical fatigue the individual experiences from dressing themselves independently might mean they have limited energy to participate in sport/exercise. The following items relate to your daily activities, to assist your health/fitness professional understand how best to support your participation in sport/exercise. You will be asked to identify if and how much each activity influences your sport/exercise participation, and if you require support to complete each activity. **If you believe your sport and exercise participation has not been impacted by your participation in your daily activities, please go directly to sport/exercise participation (S1).**

|  | **Please identify to what degree the following daily activities influence your sport/exercise participation.** | **1**  **Does not influence sport/**  **exercise participation** | **2**  **Slightly influences sport/**  **exercise participation** | **3**  **Influences sport/**  **exercise participation** | **4**  **Greatly influences sport/**  **exercise participation** | **5**  **Significantly influences sport/**  **exercise participation** | **I require support from a person** | **I require support from Equipment** | **I require activity adaptation** |
| --- | --- | --- | --- | --- | --- | --- | --- | --- | --- |
| **D1** | **Getting where you need to go** e.g., accessing transport. |  |  |  |  |  |  |  |  |
| **D2** | **Using the toilet.** |  |  |  |  |  |  |  |  |
| **D3** | **Cleaning yourself.** |  |  |  |  |  |  |  |  |
| **D4** | **Dressing and undressing yourself.** |  |  |  |  |  |  |  |  |
| **D5** | **Eating and drinking,** e.g., preparing and eating regular meals, remembering to and drinking water throughout the day. |  |  |  |  |  |  |  |  |
| **D6** | **Managing your health** e.g., remembering and taking medication. |  |  |  |  |  |  |  |  |
| **D7** | **Organising yourself** e.g., organising belongings before leaving the house. |  |  |  |  |  |  |  |  |
| **D8** | **Getting enough sleep.** |  |  |  |  |  |  |  |  |
| **D9** | **Managing life responsibilities** e.g., employment, education, household chores, caring for a child or pet, attending appointments. |  |  |  |  |  |  |  |  |
| **D10** | **Using technology** e.g., telephone, computer, etc. |  |  |  |  |  |  |  |  |
| **Daily activities total score** | |  | | | | | | | |
| **D11** | **Please provide any further information about how your daily activities might influence your participation in sport/exercise. *Please also use this section to provide further information on supports required.*** | | | | | | | | |
|  | *Please provide response here* | | | | | | | | |

***Sport/exercise***

The following items are factors that may influence your participation in sport/exercise. These factors might relate to the person (e.g., skills and motivations), as well as the sporting/exercise activities (e.g., the difficulty of the movements), and the environments in which they are performed in (e.g., physical, social and sensory).

|  | **Please identify how you complete the following activities, and what support you require.** | **1**  **Can easily do on my own** | **2**  **Can do on my own with some difficulty** | **3**  **Can do with support** | **4**  **Can do with significant support** | **5**  **Cannot do** | **Support from a person** | **Support from Equipment** | **Activity adaptation** |
| --- | --- | --- | --- | --- | --- | --- | --- | --- | --- |
| **S1** | **Seeing things around you** e.g., at a distance and up close. |  |  |  |  |  |  |  |  |
| **S2** | **Hearing what people say to you or sounds around you.** |  |  |  |  |  |  |  |  |
| **S3** | **Managing your emotions.** |  |  |  |  |  |  |  |  |
| **S4** | **Managing your mental health.** |  |  |  |  |  |  |  |  |
| **S5** | **Motivating yourself to participate in sport/exercise.** |  |  |  |  |  |  |  |  |
| **S6** | **Remembering and/or concentrating.** |  |  |  |  |  |  |  |  |
| **S7** | **Understanding and following instructions.** |  |  |  |  |  |  |  |  |
| **S8** | **Problem solving.** |  |  |  |  |  |  |  |  |
| **S9** | **Understanding what people say to you.** |  |  |  |  |  |  |  |  |
| **S10** | **Communicating with familiar people.** |  |  |  |  |  |  |  |  |
| **S11** | **Communicating with unfamiliar people.** |  |  |  |  |  |  |  |  |
| **S12** | **Joining group activities.** |  |  |  |  |  |  |  |  |
| **S13** | **Finding other people to participate with.** |  |  |  |  |  |  |  |  |
| **S14** | **Advocating for my own needs (including having people listen and respond to these needs).** |  |  |  |  |  |  |  |  |
| **S15** | **Getting to community activities/programs.** |  |  |  |  |  |  |  |  |
| **S16** | **Accessing and managing funding required to participate in sport/exercise.** |  |  |  |  |  |  |  |  |
| **S17** | **Performing transfers** e.g., in and out of a swimming pool, on and off equipment, etc. |  |  |  |  |  |  |  |  |
| **S18** | **Performing specific sport/exercise movement/skills.** |  |  |  |  |  |  |  |  |
| **S19** | **Using sport/exercise equipment** e.g., racquets, balls, exercise machines, etc. |  |  |  |  |  |  |  |  |
| **S20** | **Moving/balancing on flat ground.** |  |  |  |  |  |  |  |  |
| **S21** | **Moving for long distances.** |  |  |  |  |  |  |  |  |
| **S22** | **Moving/balancing on uneven ground.** |  |  |  |  |  |  |  |  |
| **S23** | **Going up and down stairs.** |  |  |  |  |  |  |  |  |
| **S24** | **Moving/coordinating around obstacles e**.g., gym equipment, other people, etc. |  |  |  |  |  |  |  |  |
| **S25** | **Managing the sensory environment** e.g., loud music, lots of people, dirt. |  |  |  |  |  |  |  |  |
| **Sport and exercise total score** | |  | | | | | | | |
| **S26** | **Do you require any of the following equipment/aides to participate in sport/exercise?** | | | | | | | | |
|  | - None - Cane/stick - Crutches - Orthoses - Splint - Manual wheelchair - Electric wheelchair - Pressure relief cushion - Protheses – lower limb - Protheses – upper limb - Rollators - Standing frame - Walking frame - Therapeutic footwear - Glasses - Hearing aide - White cane - Assistance animal - Augmentative and alternative communication - Noise cancelling technology - Hoister - Modified car/accessible public transport - Oxygen/ventilator - Commode - Sports specific equipment (please list) - Others (please list) | | | | | | | | |
| **S27** | **Do you experience any additional barriers to accessing sport/exercise activities that may not have been covered above.** | | | | | | | | |
|  | *Please provide response here* | | | | | | | | |
| **S28** | **Is there any additional information you would like to provide about your participation in sport/exercise. *Please also use this section to provide further information on supports required.*** | | | | | | | | |
|  | *Please provide response here* | | | | | | | | |
| **Total**  **score** |  | | | | | | | | |

| **Does not influence my sport/**  **exercise participation** | **Slightly influences my sport/exercise participation** | **Influences my sport/exercise participation** | **Greatly influences my sport/exercise participation** | **Significantly influences my sport/exercise participation** |
| --- | --- | --- | --- | --- |
| 1 | 2 | 3 | 4 | 5 |

| **Can easily do on my own** | **Can do on my own with some difficulty** | **Can do with support** | **Can do with significant support** | **Cannot do** |
| --- | --- | --- | --- | --- |
| 1 | 2 | 3 | 4 | 5 |
